# Use of Drastic Purgatives

**Published:** 1842-06-25

**Authors:** 


*Use of Drastic Purgatives.*—Two cases are recorded in the "Bulletin Genéral de Thérapeutique," in which the continued use of drastic purgatives cured obstinate cutaneous diseases. The first, a poor, aged woman, who had been troubled with prurigo for upwards of two years, and had been under the care of a medical man, who had directed venesection, alkaline drinks and baths, milk diet, changed afterwards for as generous diet as she could procure; lotions prepared with bitter infusions; and finally, sulphur ointment, from all of which she derived only temporary benefit; was at last ordered by another practitioner to take the tartarised antimony, two grains for a dose. The first not producing any effect, she repeated it of her own accord, when its use was followed by free and abundant stools, but no vomiting. Relief following, the dose was repeated every eight or ten days, the same purgative effect being caused, and the disease gradually disappearing. At the end of three months the patient was cured.

The subject of the second case was a woman, fifty-six years old, a patient of Andral's, at la Charité, who was cured of psoriasis by repeated doses of German brandy, a drastic cathartic, composed of jalap and scammony infused in brandy, which equally produced frequent and abundant evacuations.

Mention is made incidentally of another patient of Andral's, who, while labouring under a severe attack of angina tonsillaris, was ordered a powerful cathartic, which produced more than sixty stools, the pulse falling from 104 to 76, and the patient was cured.—*Provincial Medical Journal.* May 28, 1842.

[The physician should, however, take care lest the remedy prove worse than the disease—many individuals would certainly not bear these large doses of drastics.]

